# Compositional and Metabolic Responses of Autotrophic Microbial Community to Salinity in Lacustrine Environments

**DOI:** 10.1128/msystems.00335-22

**Published:** 2022-07-12

**Authors:** Yun Fang, Jun Liu, Jian Yang, Geng Wu, Zhengshuang Hua, Hailiang Dong, Brian P. Hedlund, Brett J. Baker, Hongchen Jiang

**Affiliations:** a Key Laboratory for Green Chemical Process of Ministry of Education, School of Environmental Ecology and Biological Engineering, Wuhan Institute of Technology, Wuhan, People’s Republic of China; b State Key Laboratory of Biogeology and Environmental Geology, China University of Geosciences, Wuhan, People’s Republic of China; c State Key Laboratory of Agricultural Microbiology, State Environmental Protection Key Laboratory of Soil Health and Green Remediation, College of Resources and Environment, Huazhong Agricultural University, Wuhan, People’s Republic of China; d Department of Environmental Science and Engineering, University of Science and Technology of China, Hefei, People’s Republic of China; e State Key Laboratory of Biogeology and Environmental Geology, China University of Geosciences, Beijing, People’s Republic of China; f School of Life Sciences, University of Nevada Las Vegas, Las Vegas, Nevada, USA; g Department of Marine Science, Marine Science Institute, University of Texas Austin, Port Aransas, Texas, USA; h Department of Integrative Biology, University of Texas at Austin, USA; i Qinghai Provincial Key Laboratory of Geology and Environment of Salt Lakes, Qinghai Institute of Salt Lakes, Chinese Academy of Sciences, Xining, People’s Republic of China; California State University, Northridge

**Keywords:** autotrophic microorganisms, salinity, genome-resolved metagenomics, carbon fixation pathway, saline lakes

## Abstract

The compositional and physiological responses of autotrophic microbiotas to salinity in lakes remain unclear. In this study, the community composition and carbon fixation pathways of autotrophic microorganisms in lacustrine sediments with a salinity gradient (82.6 g/L to 0.54 g/L) were investigated by using metagenomic analysis. A total of 117 metagenome-assembled genomes (MAGs) with carbon fixation potentially belonging to 20 phyla were obtained. The abundance of these potential autotrophs increased significantly with decreasing salinity, and the variation of sediment autotrophic microbial communities was mainly affected by salinity, pH, and total organic carbon. Notably, along the decreasing salinity gradient, the dominant lineage shifted from *Desulfobacterota* to *Proteobacteria*. Meanwhile, the dominant carbon fixation pathway shifted from the Wood-Lungdahl pathway to the less-energy-efficient Calvin-Benson-Bassham cycle, with glycolysis shifting from the Embden-Meyerhof-Parnas pathway to the less-exergonic Entner-Doudoroff pathway. These results suggest that the physiological efficiency of autotrophic microorganisms decreased when the environmental salinity became lower. Metabolic inference of these MAGs revealed that carbon fixation may be coupled to the oxidation of reduced sulfur compounds and ferrous iron, dissimilatory nitrate reduction at low salinity, and dissimilatory sulfate reduction in hypersaline sediments. These results extend our understanding of metabolic versatility and niche diversity of autotrophic microorganisms in saline environments and shed light on the response of autotrophic microbiomes to salinity. These findings are of great significance for understanding the impact of desalination caused by climate warming on the carbon cycle of saline lake ecosystems.

**IMPORTANCE** The Qinghai-Tibetan lakes are experiencing water increase and salinity decrease due to climate warming. However, little is known about how the salinity decrease will affect the composition of autotrophic microbial populations and their carbon fixation pathways. In this study, we used genome-resolved metagenomics to interpret the dynamic changes in the autotrophic microbial community and metabolic pathways along a salinity gradient. The results showed that desalination drove the shift of the dominant microbial lineage from *Desulfobacterota* to *Proteobacteria*, enriched autotrophs with lower physiological efficiency pathways, and enhanced coupling between the carbon cycle and other element cycles. These results can predict the future response of microbial communities to lake desalination and improve our understanding of the effect of climate warming on the carbon cycle in saline aquatic ecosystems.

## INTRODUCTION

Global climate warming is one of the greatest scientific and policy concerns in this century, and it has already triggered changes in ecosystem structure, processes, and functions (1, 2). Carbon dioxide is a major greenhouse gas contributing to global temperature increases of both land and ocean surfaces (3). The flow of carbon dioxide from atmosphere to biosphere is through autotrophic carbon fixation. Autotrophic carbon fixation underlies nearly all biological processes on Earth (4), and autotrophic microbes in aquatic ecosystems (dominated by cyanobacteria and microalgae) provide approximately 50% (~50 Pg C year^−1^) of the annual global net primary production (5, 6). Sedimentary microbial autotrophs play a key role in the carbon cycle of aquatic ecosystems, participating in energy transfer and nutrient cycle turnover (7). Different types of sedimentary microbial autotrophs have different carbon dioxide and nutrient (e.g., N, S, and Fe) utilization capacities. They could reduce nutrient concentrations in the sediment pore water, cause large fluctuations in both oxygen and dissolved inorganic carbon concentrations, and affect both pH and the redox potential (7). Thus, it is of great importance to understand carbon fixation mechanisms and physiologies of autotrophic microbial communities in the sediments of aquatic habitats.

To date, eight pathways have been confirmed to fix inorganic carbon by autotrophic organisms, including the Calvin-Benson-Bassham (CBB) cycle, reductive citric acid (rTCA) cycle, Wood-Ljungdahl (WL) pathway, 3-hydroxypropionate (3-HP) bicycle, 3-hydroxypropionate/4-hydroxybutyrate (HP/HB) cycle, dicarboxylate/hydroxybutyrate (DC/HB) cycle, reductive glycine (rGly) pathway, and reversed oxidative tricarboxylic acid (roTCA) cycle (8, 9). In previous community-level studies, markers for carbon fixation (such as *rbcL* and *aclB*) were leveraged to explore the composition and diversity of potential autotrophs (10, 11). However, recent studies have shown that form IV and IV-like RubisCOs encoded by *rbcLS* genes may perform functions in methionine salvage, sulfur metabolism, and d-apiose catabolism rather than carbon fixation (12, 13). Therefore, the identification of autotrophic pathways based on single genes may be inaccurate. Moreover, autotrophs can perform other important ecological functions, such as nitrogen fixation, iron and sulfur oxidation, and hydrogen utilization, that are not revealed by studies of single genes. Reconstruction of individual genomes from nature through genome-resolved metagenomics is more informative because it can provide deeper and more accurate insights into carbon fixation potential and potential coupling between autotrophic pathways and other element cycles (e.g., N, S, and Fe) at a community level.

**FIG 1:**
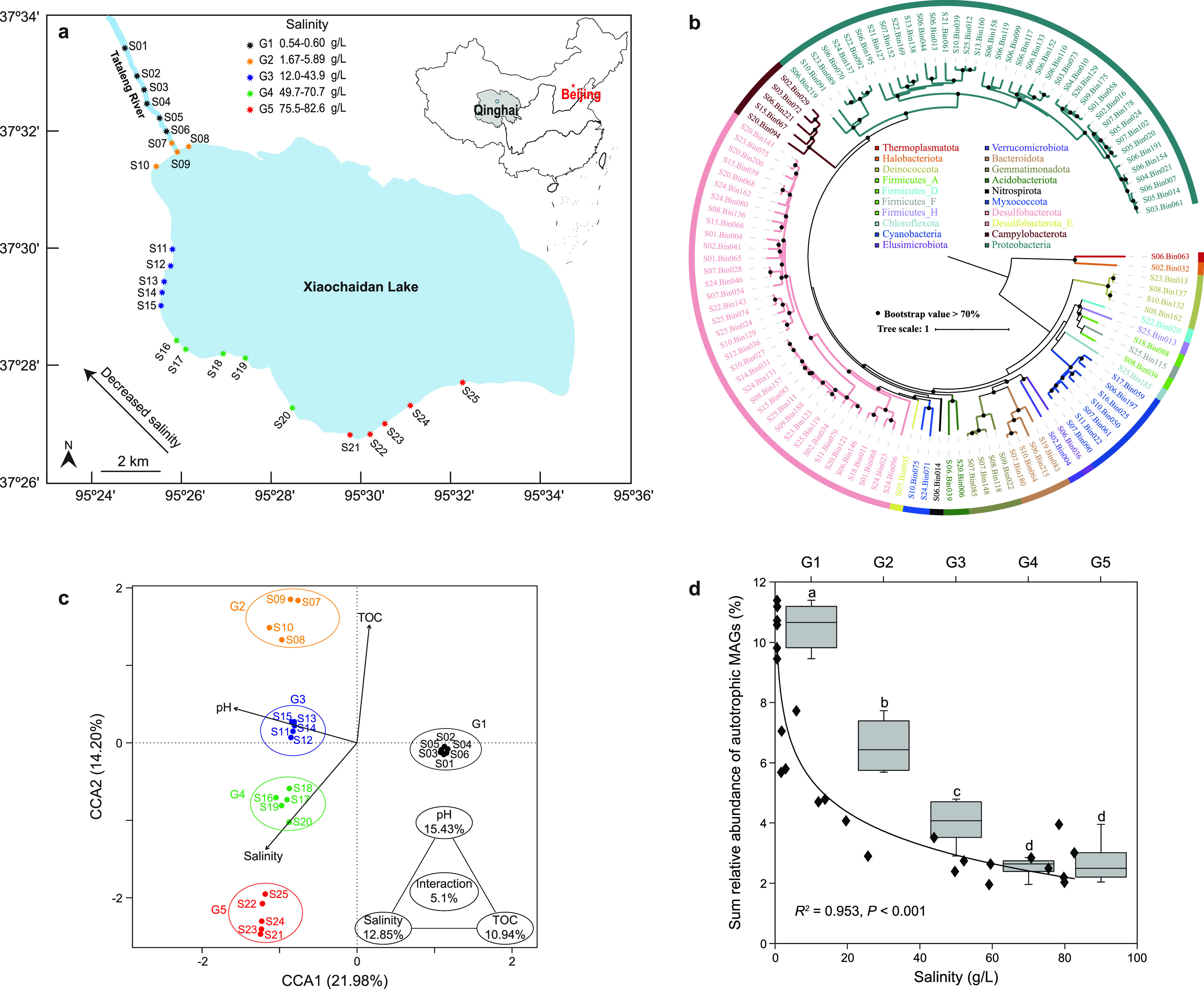
Properties of potential carbon fixers in lacustrine sediments. (a) Geographic location of 25 sediment samples collected from Xiaochaidan Lake and its inflow Tataleng River. (b) Phylogenetic placement of the 117 retrieved MAGs. The phylogenomic tree was constructed using the concatenated alignment of 31 marker genes. Bootstrap values are based on 1,000 replicates, and percentages of >70% are shown with black circles. Phylogenomic analyses of archaeal and bacterial MAGs using the GTDB database are shown in Fig. S1a and b, respectively, in the supplemental material. (c) Canonical correspondence analysis of the potential carbon-fixing community and environmental variables. (d) Inverse relationship between the total relative abundance of all potential carbon-fixing microbes in each sample (black diamond and trend line) and the corresponding sediment salinity. The box plot shows the relative abundances of all potential carbon fixers in samples of each salinity group (G1 to G5). Different letters above the bars indicate a significant difference between different groups at a 5% level, according to one-way ANOVA with the Duncan test.

The Qinghai-Tibetan Plateau (QTP) is the highest and largest highland on Earth. The QTP glacier ice reserve is only less than that in the polar regions, and seasonal glacial meltwater feeds thousands of saline lakes (14, 15). Known as the “Roof of the World,” the QTP is also the most sensitive area to climatic fluctuations, where the regional warming rate of approximately 0.32°C/10 years is twice the rate of global warming during the past 3 decades (16). Climate warming has accelerated glacier retreat, increased precipitation, and decreased evaporation (17), resulting in the expansion of glacial lakes on the QTP and an overall decrease in lake salinity, a process known as lake desalination (18, 19). Numerous studies have shown salinity to be an important regulator of microbial diversity, community structure, metabolic activities, and microbial functional groups in diverse lake ecosystems (10, 15, 20), likely due to energetic constraints (21). These bioenergetic constraints determine the upper limit of salt tolerance of microbial guilds, below which different dissimilatory processes can occur in nature. A recent study explored the effect of salinity on the abundance and diversity of microbial primary producers carrying the *cbbL* gene (10), yet that study is limited because different autotrophs with distinct autotrophic pathways likely dominate in different salinity ranges. Currently, the effects of salinity on the distribution of autotrophic organisms and pathways have not been explored. To fill this knowledge gap within the context of lake desalination induced by global warming, we collected 25 sediment samples along a salinity gradient (82.6 g/L to 0.54 g/L) in Xiaochaidan Lake and its inflowing Tataleng River on the QTP (Fig. 1a). Genome-resolved metagenomic analysis and metabolic inference revealed the taxonomy and potential biogeochemical capabilities of 117 putative autotrophs in this ecosystem. Analysis of these MAGs revealed systematic trends within the salinity gradient, portending a future microbial community response to lake desalination and improving our understanding of the effect of climate warming on lacustrine microbial ecology.

10.1128/msystems.00335-22.1 FIG S1 Phylogenomic analysis of archaeal (a) and bacterial (b) MAGs using the GTDB database with 122 and 120 marker genes, respectively. Bootstrap values are based on 1,000 replicates, and percentages of >70% are shown with black circles. Download FIG S1, PDF file, 0.3 MB. Copyright © 2022 Fang et al. 2022 Fang et al. https://creativecommons.org/licenses/by/4.0/ This content is distributed under the terms of the Creative Commons Attribution 4.0 International license.

## RESULTS AND DISCUSSION

### Autotrophic communities in sediments and environmental determinants.

Approximately 2 Tb of raw reads was generated for these 25 sediment samples, and *de novo* metagenomic assembly resulted in ~10 Gb of scaffolds (≥2,000 bp) (see Data Set S1a in the supplemental material). Through genome binning, 117 metagenome-assembled genomes (MAGs) with the predicted capability of carbon fixation were selected for subsequent analysis (Data Set S1b). The sizes of these MAGs ranged widely, from ~1.1 to 7.1 Mb, with estimated completeness of 43% to 100% and <3.5% single-marker-gene redundancies (Data Set S1c). According to the criteria for assessing the quality of MAGs (22), 22 and 92 genomes in this study were defined as high-quality and medium-quality drafts, respectively. The result is likely attributed to the high diversity of sediment microbiomes in saline lakes (23). Note that these 117 MAGs varied greatly in GC content, from 30% to 74%, and of these MAGs, ~44% showed a high GC content (≥60%).

10.1128/msystems.00335-22.8 DATA SET S1 (a) Information of metagenomic data sets and assembly results. (b) Number of genes assigned to five known pathways of carbon fixation. (c) Summary of genomic characteristics of putative autotrophic microbes recovered from metagenomes. (d) Relative abundances of these putative autotrophic microbes across 25 sediments. (e) Number of genes related to carbon metabolism and respiration. (f) Number of genes assigned to sulfur metabolism. (g) Number of genes assigned to nitrogen metabolism. (h) Number of genes assigned to iron metabolism. (i) Physicochemical characteristics of sediment samples. Download Data Set S1, XLSX file, 0.3 MB. Copyright © 2022 Fang et al. 2022 Fang et al. https://creativecommons.org/licenses/by/4.0/ This content is distributed under the terms of the Creative Commons Attribution 4.0 International license.

Phylogenomic trees of these MAGs based on 31/bac120/arc122 marker gene sets showed that the MAGs were affiliated with 18 bacterial phyla and 2 archaeal phyla, including *Proteobacteria* (*n *=* *40), *Desulfobacterota* (*n *=* *38), *Cyanobacteria* (*n *=* *7), *Campylobacteria* (*n *=* *5), *Bacteroidetes* (*n *=* *4), *Deinococcota* (*n *=* *4), *Gemmatimonadota* (*n *=* *4), *Acidobacteriota* (*n *=* *2), *Firmicutes_*A (*n *=* *2), *Firmicutes_*D (*n *=* *1), *Firmicutes_*E (*n *=* *1), *Firmicutes_*F (*n *=* *1), *Desulfobacterota*_E (*n *=* *1), *Myxococcota* (*n *=* *1), *Verrucomicrobiota* (*n *=* *1), *Chloroflexota* (*n *=* *1), *Elusimicrobiota* (*n *=* *1), *Nitrospirota* (*n *=* *1), *Halobacteriota* (*n *=* *1), and *Thermoplasmatota* (*n *=* *1) (Fig. 1b and Fig. S1). Average nucleotide identity (ANI) analysis demonstrated that each MAG represented a unique species based on the species demarcation threshold of 95% (24). Notably, eight genomes did not belong to any known order, expanding the diversity of autotrophs. These findings illustrate that the potential autotrophic community in the studied sediments was taxonomically diverse. The relative abundance of these potential autotrophs in each community varied between ~2% and ~11% and showed a significant negative correlation (*R^2^* = 0.953, *P < *0.001) with salinity (Fig. 1d and Data Set S1d).

To identify environmental parameters influencing the autotrophic community, a BIOENV analysis was applied, and a subset of three variables (pH, salinity, and total organic carbon [TOC]) was selected. Canonical correspondence analysis (CCA) showed that a significant portion of the community variation was explained by these variables (*P = *0.001), with 55.7% of the variation unexplained (Fig. 1c). Variance partitioning analysis indicated that 15.4%, 12.9%, 10.9%, and 5.1% of the variation was explained by pH (*P = *0.001), salinity (*P = *0.001), TOC (*P = *0.002), and their interactions, respectively. This is consistent with numerous studies showing that salinity and pH are important determinants of microbial diversity and community structure in sediments (10, 23–26). A recent study also revealed that salinity structured the sediment bacterial community at the oligohaline-marine transition of the Baltic Sea (26). TOC also frequently shapes microbial community structure in aquatic ecosystems (27). Organic carbon could facilitate the growth of heterotrophs and facultative autotrophs, giving them a selective advantage over obligate autotrophic microbes. Moreover, high salinity exerts strong pressure on the energetics of autotrophic pathways (28), and thus the availability of TOC could make heterotrophs and facultative autotrophs even more competitive under high salinity. These direct and indirect effects are consistent with the observed variations in autotrophic community structure. A recent study demonstrated that TOC and total nitrogen (TN) contributed to the response of an autotrophic microbial community to salinity and mitigated the salinity constraints (10). In short, these physicochemical conditions exerted a critical impact on sediment autotrophic communities.

CCA and nonmetric multidimensional scaling (NMDS) analysis separated these sediment communities into five categories, and this clustering pattern matched the salinity gradient (Fig. 1c and d; Fig. S2a). This result underscores the importance of salinity in structuring these autotrophic communities, in agreement with previous findings in aquatic ecosystems (10). Unlike three salinity groups based on principal-component analysis in the previous study (10), our sediment samples were divided into five salinity groups: G1 (S01 to S06; salinity, ~0.6 g/L), G2 (S07 to S10; salinity, 1.8 to 5.9 g/L), G3 (S11 to S15; salinity, 12.0 to 43.9 g/L), G4 (S16 to S20; salinity, 49.7 to 70.7 g/L), and G5 (S21 to S25; salinity, 75.5 to 82.6 g/L). Furthermore, an abundance-salinity correlation was observed, with the abundance of these autotrophs decreasing from G1 to G5 (one-way analysis of variance [ANOVA], *P < *0.001) (Fig. 1e). This inverse correlation is likely due to two reasons: (i) salinity-derived osmotic stress directly limits the metabolic activity of autotrophs and consumes energy to maintain cellular osmotic balance, resulting in a decrease in autotrophic biomass, and (ii) high salinity decreases the solubility and diffusion coefficient of CO_2_, thus limiting its availability for carbon fixation, and finally, autotrophic biomass decreases (21, 29).

10.1128/msystems.00335-22.2 FIG S2 Diversity distribution and relative abundances of carbon-fixing community. (a) Nonmetric multidimensional scaling (NMDS) analysis of the potential carbon-fixing community. (b) Average relative abundances of different taxa in five salinity groups. (c) Shared MAGs among 25 samples belonging to five salinity groups. Each line between any two samples means one shared MAG. Download FIG S2, PDF file, 2.8 MB. Copyright © 2022 Fang et al. 2022 Fang et al. https://creativecommons.org/licenses/by/4.0/ This content is distributed under the terms of the Creative Commons Attribution 4.0 International license.

**FIG 2:**
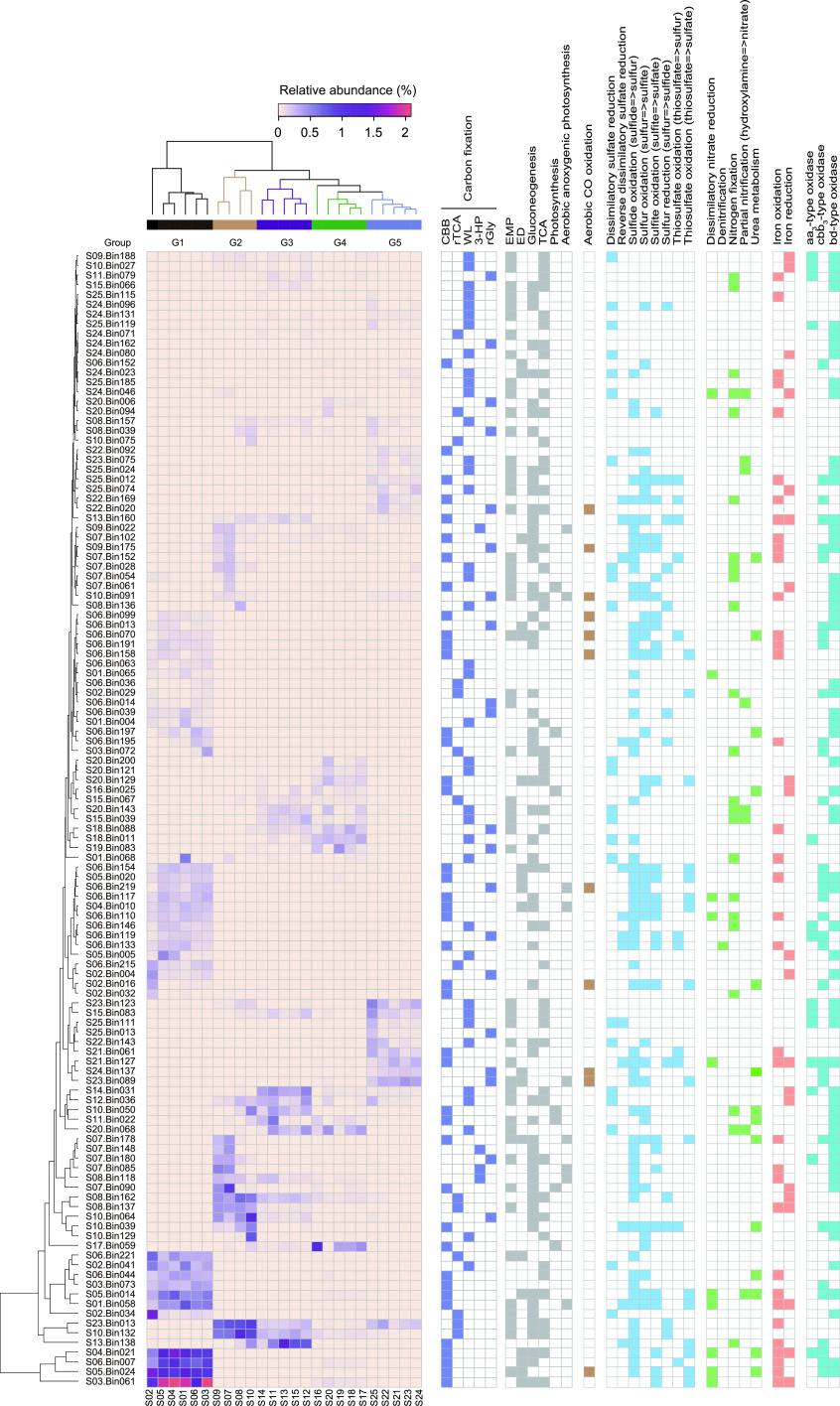
Heat map plotting the abundance pattern of 117 MAGs and presence of metabolic pathways. MAGs were organized and clustered by their relative abundance in samples, which correlated with salinity groups. The data for relative abundance of each MAG are provided in Data Set S1d, and details of metabolic pathways are provided in Data Set S1b and S1e to h. CBB, Calvin-Benson-Bassham cycle; rTCA, reductive citric acid cycle; WL, Wood-Ljungdahl pathway; 3-HP, 3-hydroxypropionate bicycle; rGly, reductive glycine pathway; EMP, Embden-Meyerhof-Parnas pathway; ED, Entner-Doudoroff pathway; TCA, citric acid cycle.

Among these five salinity groups, the autotrophic community composition was significantly different (analysis of similarity [ANOSIM], Adonis, and multiresponse permutation procedure [MRPP], all *P = *0.001). The freshwater G1 communities consisted mainly of *Proteobacteria* and *Desulfobacterota* (average relative abundances, 8.18% and 1.07%, respectively), while the *Deinococcota* (2.50%), *Proteobacteria* (0.95%), and *Desulfobacterota* (0.91%) were predominant autotrophs in the G2 communities (Fig. S2b). For the G3 to G5 groups, the *Desulfobacterota* was the main autotrophic phylum, with the average abundance between 0.86% and 1.55%. Note that autotrophic *Desulfobacterota* showed no significant difference in relative abundance among these salinity-based groups, yet the abundance of autotrophic *Proteobacteria*, *Cyanobacteria*, and *Deinococcota* varied along the salinity gradient (Fig. S2b). At the genus level, the dominant autotrophs were affiliated with *Rhodoferax* and *Hydrogenophaga* in the freshwater G1 sediments, *Planktothricoides* and *Thioalkalivibrio*_B in G2, *Desulfotignum* and *Phormidium*_A in G3, *Spirulina* and *Desulforhopalus* in G4, and *Desulfotignum* and *Maritimibacter* in the hypersaline site, G5. Intriguingly, *Desulfotignum* was always one of dominant autotrophic genera in saline to hypersaline environments (G2 to G5), in agreement with previous research that members of the *Desulfotignum* genus could adapt to a broad range of salinity (30). Furthermore, no autotrophic species were shared between the freshwater G1 and hypersaline G5 sediments (Fig. S2c). Analyses of the three most abundant species per group revealed that they were significantly more abundant in that group than in all other salinity groups (one-way ANOVA, all *P < *0.01), except *Desulfobacteraceae* S20.Bin068, inferring that these most dominant species were ecological specialists (Fig. 2 and Fig. S3).

10.1128/msystems.00335-22.3 FIG S3 Detailed heat map plotting the abundance pattern of 117 MAGs, and presence of metabolic pathways. MAGs were organized and clustered by relative abundance and salinity. The data of relative abundance for each MAG is provided in Data Set S1d, and details of metabolic pathways are provided in Data Set S1b and S1e to h. CBB, Calvin-Benson-Bassham cycle; rTCA, reductive citric acid cycle; WL, Wood-Ljungdahl pathway; 3-HP, 3-hydroxypropionate bicycle; rGly, reductive glycine pathway; PPP, pentose phosphate pathway; EMP, Embden-Meyerhof-Parnas pathway; ED, Entner-Doudoroff pathway; TCA, citric acid cycle. Download FIG S3, PDF file, 0.5 MB. Copyright © 2022 Fang et al. 2022 Fang et al. https://creativecommons.org/licenses/by/4.0/ This content is distributed under the terms of the Creative Commons Attribution 4.0 International license.

### Carbon fixation pathways in sediments.

**FIG 3:**
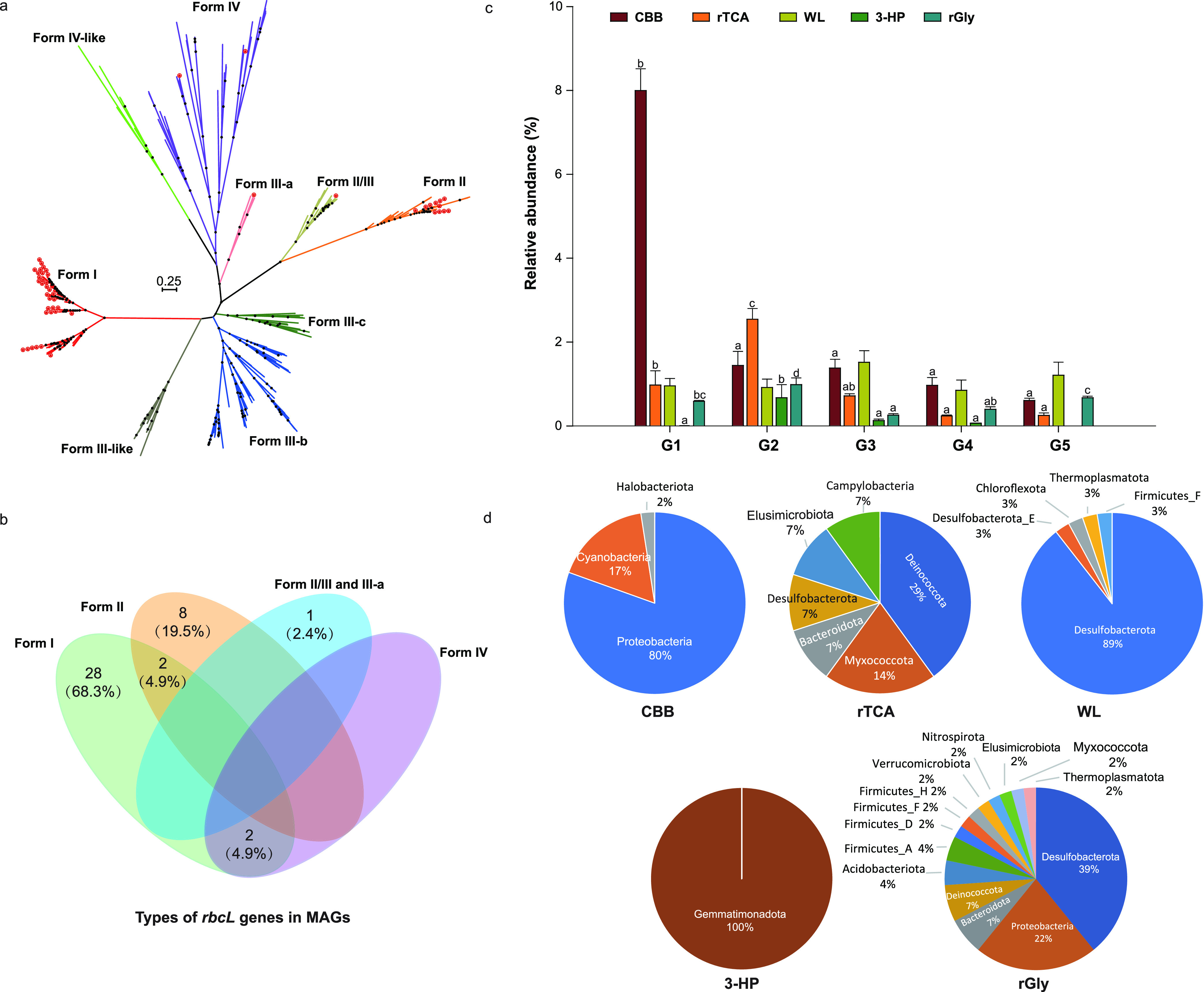
Pathways of carbon fixation identified in the 117 MAGs recovered from sediments. (a) Phylogenetic tree of the RubisCO large-subunit RbcL detected in these MAGs (with red stars). The detail of this phylogenetic tree is provided in Fig. S4. (b) Venn diagram depicting the number (percent) of the shared and unique forms of RubisCOs in these MAGs. (c) Relative abundances of MAGs with different carbon fixation pathways across five salinity groups. Different letters above the bars within a specific pathway indicate significant differences between groups at the 5% level using one-way ANOVA with the Duncan test. (d) Proportions of MAGs encoding each autotrophic pathway by phylum.

Among currently known carbon fixation pathways (8, 9), five were identified in these 117 MAGs, of which 41, 38, 20, 14, and 4 harbored the CBB, WL, rGly, rTCA, and 3-HP pathways, respectively (Fig. 2). Ribulose-1,5-bisphophate carboxylase/oxygenase (RubisCO), which is considered to be the most abundant enzyme on Earth, is integral to carbon fixation via the CBB cycle (12, 13). A phylogenetic tree of the RubisCO large subunits (RbcL) was constructed to determine the RubisCO forms present in this study (Fig. 3a and Fig. S4). Among the 41 MAGs containing the CBB cycle, almost all could encode form I or/and II RubisCOs, except *Methanothrix* sp. S02.Bin032, which encoded II/III and III-a RbcL forms. Forms III and II/III RubisCOs, primarily found in archaea, enable light-independent CO_2_ incorporation into C_5_ sugars derived from nucleotides like AMP (12, 31). Along the decreasing salinity gradient, most MAGs encoding RubisCOs were *Proteobacteria*. This is consistent with a recent study showing that *Proteobacteria* was dominant among CBB-encoding autotrophic communities in Qinghai-Tibetan lakes (10). But the dominant MAGs encoding the rTCA cycle changed from *Campylobacterota* in G1 to *Deinococcota* in G2 to G5. In contrast, the WL pathway was mainly encoded by the *Desulfobacterota* in all five salinity groups.

10.1128/msystems.00335-22.4 FIG S4 Phylogenetic tree of the RubisCO larger-subunit RbcL. Bootstrap values are based on 100 replicates, and only bootstrap values higher than 50% are indicated by circles. The circle size represents the bootstrap value. Download FIG S4, JPG file, 2.8 MB. Copyright © 2022 Fang et al. 2022 Fang et al. https://creativecommons.org/licenses/by/4.0/ This content is distributed under the terms of the Creative Commons Attribution 4.0 International license.

**FIG 4:**
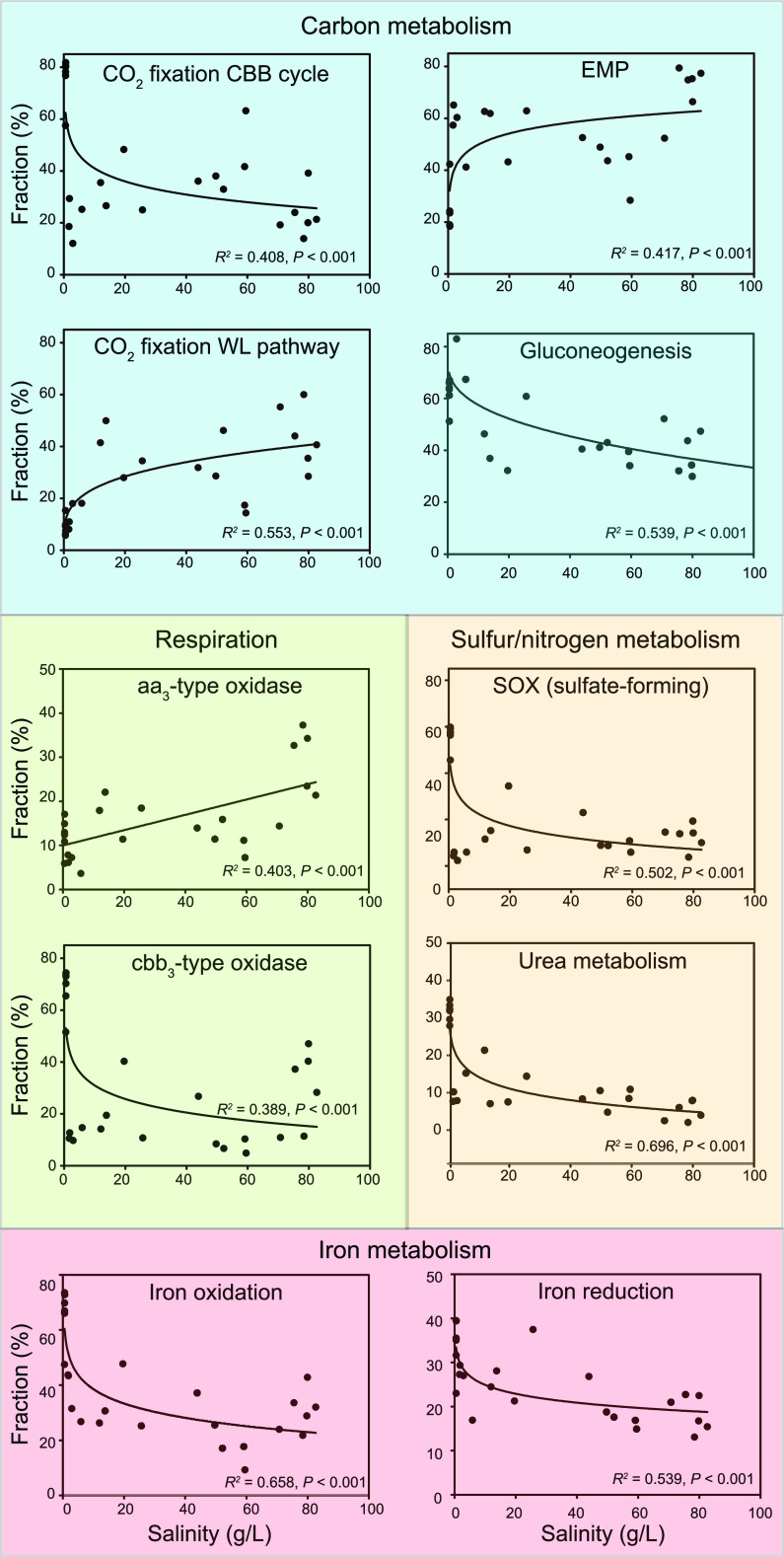
Relative fraction of potential carbon-fixing MAGs with metabolic capabilities in each sample along the salinity gradient. Curve fitting analyses in the blue, yellow, and green areas represent MAGs with carbon metabolism, sulfur/nitrogen/iron metabolism, and respiration-related metabolic potential.

Comparison of carbon fixation pathways among the five salinity groups revealed that the CBB, rTCA, and WL were the most frequent pathways in sediments, which may be attributed to their wide distribution in diverse *Bacteria* and *Archaea*. In the freshwater G1 sediments, the CBB cycle was predominant (*t* test, all *P < *0.001), and autotrophs containing this pathway showed a higher relative abundance than that of the other autotrophic groups (one-way ANOVA, *P < *0.01). The three most abundant autotrophs in G1 sediments, *Rhodoferax* sp. S03.Bin061, *Hydrogenophaga* sp. S05.Bin024, and *Burkholderiaceae* S04.Bin021, encode the CBB cycle. These results not only provided the first evidence of autotrophy in the genus *Rhodoferax* but also confirmed that members of the genus *Hydrogenophaga* can use the CBB cycle for carbon sequestration (32). In the G2 samples, the rTCA cycle dominated (*t* test, all *P < *0.001), and autotrophs encoding the rTCA were more abundant than MAGs encoding other autotrophic pathways (one-way ANOVA, *P = *0.01). In the saline to hypersaline sediments (G3 to G5), the CBB and WL pathways were dominant, with the WL being the most important pathway in the hypersaline G5 samples (Fig. 3b). Statistical analysis indicated a significant negative correlation (*P < *0.001) between the fraction of CBB-encoding MAGs and salinity (Fig. 4). In contrast, there was a significant positive association (*P < *0.001) between the fraction of the WL-encoding MAGs and salinity (Fig. 4). This correlation may be ascribed to the fact that autotrophs need to reallocate energy to accumulate inorganic and organic osmoregulators (e.g., K^+^, sugars, polyols, quaternary amines) to deal with intracellular osmotic stress (33, 34), mandating high-efficiency autotrophic pathways. High salinity results in the decrease of the solubility and diffusion coefficients of CO_2_ and O_2_ (21, 29) and is particularly stressful for autotrophic energy metabolism (28). Thus, the more energy-efficient WL pathway utilized by obligate anaerobes may provide an advantage for growth under high-salinity conditions. A recent study indicated that the CO_2_ reduction via the WL pathway was more ATP efficient and yielded more biomass than CO_2_ carboxylation-dependent pathways (including CCB, rTCA, and 3-HP) (8). The inference of an operative WL pathway in *Desulfotignum* sp. S23.Bin123 and S15.Bin083, dominant in the G5 group, was supported by previous research (35). Overall, these pathway shifts along the salinity gradient suggest adaptive advantages of different carbon fixation mechanisms as a response to salinity.

### Metabolic potential.

To understand the biogeochemical roles of autotrophic microbes, we reconstructed metabolic pathways of these 117 MAGs (Fig. 2 and Data Set S1e to h). Results revealed that approximately 56% of them harbored glycolytic pathways, including Embden-Meyerhof-Parnas (EMP; *n *=* *51) and Entner-Doudoroff (ED; *n *=* *19), while five species (*Hydrogenophaga* sp. S05.Bin024 and S07.Bin178, *Maritimibacter* sp. S23.Bin089, *Sulfurovum* sp. S06.Bin221, and *Tabrizicola* sp. S06.Bin070) contained both pathways. Previous studies have shown that some bacteria encode both EMP and ED pathways, and such bacteria usually possess incomplete pentose phosphate pathways (PPP) (36, 37). In contrast, in this study, *Hydrogenophaga* sp. S07.Bin178 was an exception, because it contained all genes encoding the complete PPP. Further statistical analyses showed a significantly positive trend (*P < *0.001) between the fraction of the EMP-encoding autotrophic MAGs and salinity (Fig. 4). This is likely due to the higher free energy yield of the fermentative EMP pathway from C_6_ and C_5_ sugars than of the ED pathway (38), and the additional energy generated is beneficial for autotrophs to resist salinity stress. Hence, autotrophs with the EMP pathway would be enriched under high salinity.

Moreover, roughly 50% of the obtained 117 MAGs had the capacity for gluconeogenesis, and the proportion of MAGs with this potential showed a significant linear positive correlation (*P < *0.001) with TOC (Fig. S5). This correlation can be explained by the fact that high TOC content inhibits carbon fixation, selecting for the capacity to produce glucose from organic precursors (10, 28). To conserve energy, many (especially facultative) autotrophs utilize organic matter to produce glucose via gluconeogenesis (10, 28). Besides, the proportion of MAGs with gluconeogenesis potential showed a significantly negative correlation with salinity (*P < *0.001). Under increased salinity, autotrophs need to consume extra energy to resist salt stress, and gluconeogenesis is an energy-consuming process, so autotrophs with the gluconeogenesis capability are inhibited.

10.1128/msystems.00335-22.5 FIG S5 Relationship between total organic carbon (TOC) and the relative fraction of potential carbon-fixing MAGs with gluconeogenesis in each sample. Download FIG S5, PDF file, 0.4 MB. Copyright © 2022 Fang et al. 2022 Fang et al. https://creativecommons.org/licenses/by/4.0/ This content is distributed under the terms of the Creative Commons Attribution 4.0 International license.

Eleven MAGs were identified with *coxLMS* genes encoding aerobic CO dehydrogenase (such as the dominant *Hydrogenophaga* sp. S05.Bin024, *Desulfotignum* sp. S14.Bin031, S15.Bin083, and S23.Bin123, and *Maritimibacter* sp. S23.Bin089), indicating that they are putative aerobic CO oxidizers. Previous studies also confirmed that members of the genera *Hydrogenophaga* and *Desulfotignum* could oxidize CO and generate reducing equivalents for nitrate reduction (32, 37). Thus, it is reasonable to hypothesize that these 11 MAGs may have the potential to oxidize CO formed from photochemical degradation of dissolved organic carbon in aquatic environments and then use the generated electrons to support ATP generation and CO_2_ fixation. This CO oxidation potential was present in autotrophic MAGs at highest frequency in the hypersaline G5 sediments (*t* test, all *P < *0.01).

Furthermore, all seven cyanobacterial MAGs contained genes for oxygenic photosynthesis (*psa* or *psb*). Alternatively, *pufABLM* genes were detected in several MAGs affiliated with *Proteobacteria* (*n *=* *6) and *Gemmatimonadota* (*n *=* *3), indicating that they are potential aerobic anoxygenic photosynthetic (AAP) bacteria (39). Three proteobacterial MAGs (*Burkholderiaceae* S04.Bin010, *Rubrivivax* sp. S01.Bin058, and *Hydrogenophaga* sp. S07.Bin178) carried the potential to perform aerobic anoxygenic photosynthesis and the CBB cycle, suggesting that RuBisCO in AAP bacteria was involved in both CO_2_ fixation and the central redox cofactor recycling, because CO_2_ fixation could be used to maintain redox balance by recycling reduced redox cofactors for photoheterotrophic metabolism (40).

### (i) Aerobic respiration.

Genes encoding the aa_3_- and cbb_3_-type cytochrome *c* oxidases (*coxABCD* and *ccoNOQP*, respectively) and cytochrome *bd* ubiquinol oxidase (*cydAB*) for aerobic respiration were found in many of the MAGs (*n *=* *14, 33, and 59, respectively) (Data Set S1e), indicating that most of the putative autotrophs likely utilize oxygen as a terminal electron acceptor. In comparison with the low-oxygen-affinity aa_3_-type oxidase induced under oxic conditions, the cbb_3_-type and bd oxidases are high-affinity terminal oxygen reductases capable of functioning under microoxic to anoxic conditions (41), and the bd oxidase has a lower energetic efficiency than the heme-copper oxidases (aa_3_ and cbb_3_ type) as it does not pump protons (42). Given the presence of the *ccoNOQP* or/and *cydAB* genes, ~56% of these potential autotrophs were microaerophiles. Besides, a few autotrophs affiliated with the *Proteobacteria* (*n *=* *12) and *Campylobacterota* (*n *=* *1) probably adapted to a broad oxygen concentration environment with both low-oxygen-affinity (*coxABCD*) and high-affinity terminal oxygen reductases genes (*ccoNOQP* or/and *cydAB*) according to previous reports (41, 42).

### (ii) Sulfur metabolism.

**FIG 5:**
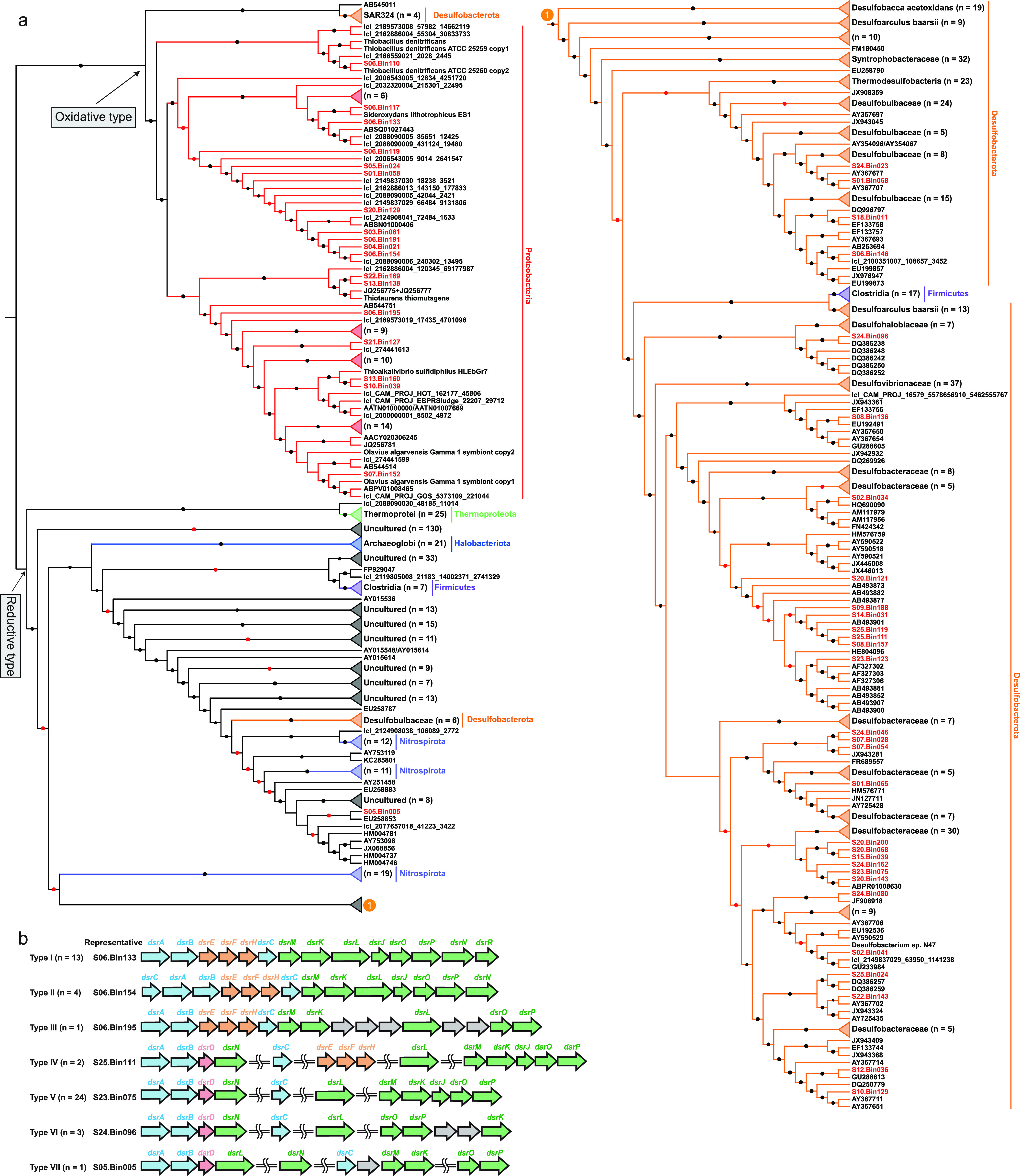
*dsr* operons of MAGs with carbon fixation potential. (a) Phylogenetic analysis of the concatenated DsrAB proteins. The detailed tree is provided in Fig. S6. Bootstrap values were based on 100 replicates, and only bootstrap values higher than 50% are shown, with red (between 50% and 75%) and black (≥75%) circles. (b) The *dsr* operon structure in 48 putative autotrophic MAGs. The number of MAGs containing a specific *dsr* operon structure is shown within parentheses.

Recent studies have revealed the important roles of autotrophs in the biogeochemical sulfur cycle (43). We found *dsrAB* genes encoding dissimilatory sulfite reductase in 49 MAGs (Data Set S1f). A phylogenetic analysis of concatenated DsrAB proteins indicates 31 reductive and 18 oxidative bacterial types (Fig. 5a and Fig. S6). Seven types of *dsr* operons in 48 of these 49 MAGs are summarized here (Fig. 5b). Several recent studies reported that the gene composition (*dsrAB* and *dsrD*/*dsrEFH*) of the *dsr* operon might determine the direction of the dissimilatory pathway between sulfite and sulfide (43, 44). Therefore, 23 desulfobacterotal MAGs were inferred to reduce sulfate to sulfide via the dissimilatory sulfate reduction (dsr) pathway, and 13 MAGs affiliated with the *Proteobacteria* (*n *=* *12) and *Desulfobacterota* (*n *=* *1) likely oxidize sulfide to sulfate through the reverse dsr (rdsr) pathway. Moreover, only *Desulfotignum* sp. S25.Bin111 had the potential to perform both functions, likely depending on oxygen concentration and/or oxidation reduction potential (44). Notably, previous research revealed that some sulfate reducers can oxidize acetate through the oxidative WL pathway (45); thus, we could not rule out the possibility that potential sulfate-reducing bacteria (SRBs) containing the WL pathway may not perform carbon fixation, but perform a reverse function in a given environment. In contrast to previous conclusions that the *Desulfotignum* species were inferred to be sulfate-reducing bacteria (35), our results suggested that some of them might be both sulfate reducers and sulfide oxidizers, increasing our understanding of ecological roles of the genus *Desulfotignum.* In the freshwater G1 sediments, MAGs with the potential for dissimilatory sulfate reduction were the least prominent, while their counterparts with dissimilatory sulfide oxidation potential were the most prominent (*t* test, all *P < *0.05), in good agreement with the lowest concentration of sulfate and the capacity for the rdsr pathway in the dominant autotrophic MAGs in G1 sediments (*Rhodoferax* sp. S03.Bin061, *Hydrogenophaga* sp. S05.Bin024, and *Burkholderiaceae* S04.Bin021). Although *Rhodoferax* and *Hydrogenophaga* species were demonstrated to oxidize reduced sulfur compounds in many studies (37), our findings provide a new oxidation mechanism, the rdsr pathway, in these genera and strengthen their importance in the sulfur cycle.

10.1128/msystems.00335-22.6 FIG S6 Phylogenetic tree of the concatenated DsrAB proteins. Bootstrap values are based on 100 replicates, and only bootstrap values higher than 50% are indicated by red (between 50% and 75%) and black (≥75%) circles. Download FIG S6, PDF file, 1.0 MB. Copyright © 2022 Fang et al. 2022 Fang et al. https://creativecommons.org/licenses/by/4.0/ This content is distributed under the terms of the Creative Commons Attribution 4.0 International license.

The complete SOX (sulfur oxidation) system was detected in 16 MAGs (Data Set S1f), mainly present in the freshwater G1 sediments (*t* test, all *P < *0.001), implying that they are able to oxidize S_2_O_3_^2−^ to SO_4_^2−^. In addition, we observed partial SOX systems (lacking *soxCD* genes) in another 11 MAGs, suggesting oxidation of S_2_O_3_^2−^ to S(0) without further oxidation of S(0) to SO_4_^2−^ (46). Among the above-mentioned 11 MAGs, four *Gammaproteobacteria*, including Thioalkalivibrio paradoxus S10.Bin039, Thioalkalivibrio nitratireducens S13.Bin160, *Thioalkalivibrio* sp. S25.Bin012, and *Gammaproteobacteria* S21.Bin127, also possessed the capacity to convert S(0) to S^2−^ via oxygenase/reductases (Sor) and sulfhydrogenases (HydGBAD) or SO_3_^2−^ by Sor and sulfur dioxygenase (Sdo). Inconsistent with previous conclusions that the *Thioalkalivibrio* species were sulfur-oxidizing bacteria (47), our findings inferred that these organisms might be sulfur reducers as well. Notably, more than a half (~52%) of the 117 MAGs contained *sqr* and/or *fccAB* genes (encoding sulfide:quinone oxidoreductase and sulfide dehydrogenase, respectively) and thus might oxidize sulfide to elemental sulfur under anaerobic conditions (48). Additionally, 44 MAGs, mainly present in the freshwater G1 sediments (*t* test, all *P < *0.05), likely utilize S(0) as an electron donor and produce SO_3_^2−^ by sulfur dioxygenase. In brief, these findings indicated a coordination in sulfur cycling among sediment autotrophs.

### (iii) Nitrogen metabolism.

To better understand the role of sediment autotrophic microbes in nutrient cycling, we reconstructed key nitrogen utilization pathways (Data Set S1g). This revealed that 23 MAGs, most prevalent in the moderate-salinity G3 sediments (*t* test, all *P < *0.05), possessed the *nifDKH* genes encoding nitrogenase, suggesting that they were autotrophic diazotrophs. In previous studies, the fixation of inorganic carbon and nitrogen by such microorganisms was recognized as a crucial process for community assembly in extreme environments (49). In the moderate-salinity G3 sediments, autotrophic communities were dominated by diazotrophs, which was also found in some Tibetan soils (50), and the reason for this phenomenon is still mysterious. The capacity of dissimilatory nitrate reduction (*napAB*, *narGHI*, and *nrfAH*/*nirBD*) was inferred in a few MAGs affiliated with the *Proteobacteria* (*n *=* *8) and *Desulfobacterota* (*n *=* *2), with the highest fraction of such microorganisms in the freshwater G1 sediments (Fig. S7). This implies that with lake desalination, carbon fixation may more closely couple with dissimilatory nitrate reduction. More than half of the MAGs (*n *=* *59) obtained in this study had some potential capacity for denitrification (*nirS*/*K*, *norBC*, and *nosZ*), but only *Rhodocyclaceae* S06.Bin133 encoded a complete denitrification pathway (nitrate to N_2_), whereas four species affiliated with the *Alphaproteobacteria* (*Rhodobacteraceae* S24.Bin137) and *Gammaproteobacteria* (*Sedimenticolaceae* S13.Bin138, S22.Bin169, and *Beggiatoaceae* S06.Bin195) encode partial pathways for nitrite reduction to nitrogen. This suggests that denitrification among sediment autotrophs is coordinated. Autotrophic nitrate-reducing and denitrifying bacteria are common in sediments (51), and the coupled processes of carbon fixation, nitrate reduction, and oxidation of reduced sulfur compounds performed by these microorganisms have been successfully applied to wastewater treatment (52). Urease genes (*ureDABCEFG*) were identified in 14 MAGs (Fig. 2), suggesting that these species could acquire C and N by hydrolyzing urea to ammonia and carbon dioxide, which then could be fixed to yield glucose via a carbon fixation pathway. The number of species and the proportion of these potential urea utilizers among autotrophs increased with decreasing salinity (both *P < *0.001) (Fig. 4). Two abundant species in the freshwater G1 sites, *Hydrogenophaga* S05.Bin024 and *Burkholderiaceae* S04.Bin021, possess the capability to utilize urea, consistent with previous research (53). Additionally, 42 MAGs may participate in partial nitrification (converting hydroxylamine to nitrate) and cooperate with other nitrifiers. In general, autotrophic prokaryotes in sediments may use a variety of strategies to obtain nitrogen or couple nitrogen transformation to growth.

10.1128/msystems.00335-22.7 FIG S7 Number count and relative fraction of potential carbon-fixing MAGs with metabolic capabilities in different salinity groups. Different letters above the bars within a specific pathway indicate significant differences between groups at the 5% level using one-way ANOVA with the Duncan test. Download FIG S7, PDF file, 0.5 MB. Copyright © 2022 Fang et al. 2022 Fang et al. https://creativecommons.org/licenses/by/4.0/ This content is distributed under the terms of the Creative Commons Attribution 4.0 International license.

### (iv) Iron metabolism.

Iron is a transition metal with redox activity that is widely present in sedimentary systems, serving as an essential nutrient and an important electron donor/acceptor to many microbes (54). In the present study, 36 MAGs were found to contain *iro*, *foxEY*, *cyc2* (cluster 1), and/or *cyc1* genes, indicating they were potential iron oxidizers (Data Set S1h) (55). This finding suggests that iron-oxidizing autotrophs have important ecological significance in surface sediments. Two-thirds of these 36 MAGs also carry the genetic potential to encode diverse terminal oxidases (Fig. 2), suggesting that the electrons are transferred to different terminal electron acceptors during iron oxidation (56). Meanwhile, 8 of these 36 MAGs have the capability to catalyze nitrate-dependent Fe(II) oxidation (NDFO) under anaerobic conditions. NDFO has been observed in natural sediments and verified by experiments (57). The presence of NDFO revealed the close coupling of Fe and N redox cycles in anaerobic sediment environments, which is of great significance to the mechanisms of NO_3_^−^ removal and the regeneration of active Fe(III) oxides in aquatic sediments, as well as the transformation of various natural and artificial organic and inorganic pollutants (54). It was notable that the proportion of MAGs with iron oxidation potential was significantly negatively associated with salinity (*P < *0.001), suggesting that C and Fe coupling is tighter under low-salinity conditions, which may increase due to lake desalination caused by climate warming. In the freshwater sediments (G1), the dominant species *Rhodoferax* S03.Bin061 showed genetic potential for iron oxidation and iron reduction due to the occurrence of *iro* and *foxEY* genes and an *mtrCAB* operon, suggesting that *Rhodoferax* species may not only be Fe(III) reducers (58) but Fe(II) oxidizers as well.

In addition to iron oxidation, 20 MAGs harbor the potential to reduce iron because they coded for ferric-chelate reductase (*feR*) and homologs of DFE_0461-0465 and MtrCAB (55, 59), of which six are both potential iron oxidizers and reducers. FeR was first characterized in a strictly anaerobic sulfate-reducing archaeon, Archaeoglobus fulgidus, which could catalyze ferric iron reduction with NAD(P)H as the electron donor (60). Intriguingly, among the *ferR*-like gene-containing MAGs (*n *=* *10), four desulfobacterial species (*Desulfotignum* S09.Bin188 and S14.Bin031 and *Desulfobacterales* S24.Bin046 and S24.Bin080) and one DFE_0461-0465-containing desulfobacterial MAG, S12.Bin036, may also have the potential to perform dissimilatory sulfate reduction. MtrCAB was shown to form a complex that transfers electrons across the outer membrane from periplasmic electron carriers to cytochrome *c* located in the outer membrane on the exterior surface and, finally, to Fe(III). Here, six proteobacterial MAGs with *mtrCAB* genes might participate in dissimilatory iron reduction (60). Additionally, seven other MAGs may oxidize or reduce iron due to the presence of a *mtrB*/*pioB*-like gene encoding decaheme-associated outer membrane proteins of the MtrB/PioB family and/or the cyc2 (cluster 3) gene on the genome, which are common among Fe(II) oxidizers and Fe(III) reducers (60). These findings imply that autotrophic microorganisms may be important players in the biogeochemical cycle of iron (especially iron oxidation) in sediment ecosystems.

### Conclusions.

Studies on the variation of autotrophic microbial population composition and potential metabolic pathways with salinity are helpful to understand the future impacts of desalination caused by global warming. In this study, variations in the autotrophic microbial community were significantly correlated with salinity, pH, and TOC. The autotrophs shifted from *Desulfobacterota* to *Proteobacteria* as salinity decreased. The predominant carbon fixation pathways shifted from the WL pathway in hypersaline sediments to the less-energy-efficient CBB cycle in freshwater sediments. Inference of the potential physiology of the autotrophic MAGs revealed that autotrophs with lower physiological efficiency would be selected by low salinity and that with the decrease of salinity, carbon fixation was more closely linked with dissimilatory nitrate reduction and oxidation of reduced sulfur compounds and ferrous iron. In addition, approximately 7% of the retrieved MAGs belonged to unknown orders, suggesting that autotrophs may be more phylogenetically and functionally diverse in saline systems than expected. The results of this study are helpful for us to understand the response of autotrophic microbiota to salinity and to predict the impact of desalination caused by climate warming on the carbon cycle in QTP lakes.

## MATERIALS AND METHODS

### Site description, sampling, geochemical measurements, and DNA extraction and sequencing.

Xiaochaidan Lake (37°27′–37°31′N, 95°25′–95°35′E) is a saline terminal lake of the sodium sulfate subtype brines near the northeastern edge of the Qaidam Basin (Fig. 1a) (14). During the past 40 years, the surface area of Xiaochaidan Lake has experienced expansion, reduction, and expansion again to its current size of 103.94 km^2^ with a maximum depth ~2.2 m (61, 62). This lake has no outlet but is fed by an inflowing freshwater river, Tataleng River, at its northwestern margin, and groundwater. In November 2016, 25 surface sediment samples (0 to 5 cm) were collected at ~1.0 m water depth with an average grain size of 5.3 to 31.8 μm (62), along the shoreline, from the Tataleng River in the northwestern corner to the southeastern margin of the lake. A sterile spade, washed with ethanol and *in situ* lake water before use, was used to collect sediments into 50-mL sterile centrifuge tubes. The samples for DNA extraction were immediately frozen in dry ice until return to the laboratory and then stored at −80°C before further analysis.

Sediment pore water was collected by centrifugation (5,000 × *g*, 10 min, 4°C) and was used to measure pH and salinity with a PP-20 meter (Sartorius, Germany). The concentrations of major ions (e.g. Na^+^, K^+^, Ca^2+^, Mg^2+^, Cl^−^, and SO_4_^2−^) were determined by using a Dionex DX 600 ion chromatograph (Dionex, USA). Additionally, total organic carbon (TOC) and dissolved organic carbon (DOC) were measured with a Multi N/C 2100S analyzer (Analytik Jena, Germany). Physicochemical characteristics are summarized in Data Set S1i in the supplemental material.

For the 25 sediment samples, genomic DNA was extracted with our modified phenol-chloroform method (63). The quality and quantity of extracted DNA were checked by using agarose gel electrophoresis and a NanoDrop 2000 spectrophotometer, respectively. Standard shotgun libraries of 300-bp insert size were prepared at the Guangdong Magigene Company and were sequenced on an Illumina HiSeq 4000 platform (paired-end 150-bp mode).

### Genome-resolved metagenomic analysis.

All raw reads were dereplicated using an in-house perl script, and the resulting unique reads were trimmed based on quality scores using Sickle (version 1.33) with the parameters “-q 20 -l 50”. The 25 individual samples were each assembled *de novo* to obtain 25 separate assemblies using SPAdes (version 3.11.0) with the parameters “-k 21, 33, 55, 77 –meta” (64). For each sample, the scaffold coverage was calculated by mapping the qualified reads to the assembled scaffolds (length, ≥2,000 bp) with BBMap. These scaffolds were clustered into MAGs based on their tetranucleotide frequencies and read coverage using MetaBAT 2 with the parameters “-m 2000 –unbinned” (65).

These MAGs were further used to call genes with the single mode of Prodigal (version 2.6.3) (66), and these predicted genes were annotated against the Pfam, KEGG, eggNOG, and NCBI-nr databases. According to the above results, genomes containing all key genes of any carbon fixation pathway were aggregated, dereplicated, and optimized using dRep (version 2.0.5) (67) and ESOM (emergent self-organizing map). For obtaining high-quality genomes, the best representative from each cluster was further optimized and reassembled as previously described (44).

Next, the optimized genome bins were evaluated for taxonomic assignment, genome completeness, potential contamination, and strain heterogeneity using CheckM (version 1.0.8) (68). The predicted gene functions were manually curated and modified by comparison to the NCBI-nr, KEGG, eggNOG, Pfam, and in-house databases. Genes involved in iron transport and metabolisms were annotated using FeGenie (44). Subsequently, the metabolic pathways of each bin were constructed based on gene annotation. The 16S rRNA gene sequences identified by CheckM were used to check the genomic taxonomy (described below) by searching for closely related sequences in NCBI GenBank with BLASTn. Lastly, calculation of relative abundances of these genomes in the sediment communities is described in the supplemental material (Text S1).

10.1128/msystems.00335-22.9 TEXT S1 Supplementary methods. Download Text S1, DOCX file, 0.02 MB. Copyright © 2022 Fang et al. 2022 Fang et al. https://creativecommons.org/licenses/by/4.0/ This content is distributed under the terms of the Creative Commons Attribution 4.0 International license.

### Phylogenetic analyses.

Phylogeny of the retrieved 117 genomes was assessed based on a concatenated alignment of 31 marker genes (69). Marker proteins were identified in these genomes with AMPHORA2 (version 3.1b1) using hidden Markov models. Each of the 31 marker protein sequences was aligned using MAFFT (version 7.313) with the parameters “–localpair –maxiterate 1000” (70), and subsequently filtered with trimAl 1.4 to remove columns comprised of ≥95% gaps. Then, the curated alignments were concatenated, and a phylogenetic tree was built using IQ-TREE (version 1.6.10) with the parameters “LG+R8 -alrt 1000 -bb 1000” (71). In addition, the Genome Taxonomy Database Toolkit (GTDB-Tk) was used to assign taxonomy to these genomes. For the phylogenetic analysis of functional marker proteins (RbcL and DsrAB), the respective protein sequences were collected from previous studies (13, 44). Alignment and filtering were performed with the same programs as described above. Phylogenetic trees were constructed using IQ-TREE with the parameters “-m TEST -alrt 1000 -bb 1000”. The newick files with the best tree topology were uploaded to iTOL for visualization and formatting.

### Statistical analyses.

The fraction of potential autotrophs with a given function as a fraction of the total potential carbon-fixing community was determined by:

$$\frac{\sum^{\;}\text{total relative abundance of potential carbon fixers with function }X}{\sum^{\;}\text{total relative abundace of potential carbon fixing community}}$$

All statistical analyses were implemented using SPSS 18.0, Origin 9.0, SigmaPlot 10.0, and various R packages (http://www.r-project.org). The relationship between the potential autotrophic community and environmental variables was analyzed using canonical correspondence analysis (CCA), while variance partitioning analysis was applied to determine the independent contributions of these environmental factors to the variation in community composition, and an unconstrained nonmetric multidimensional scaling (NMDS) analysis was used to show the separation of the potential carbon-fixing community. The significance of differences in the number of species and the fractions of potential carbon fixers with specific functions among five groups were tested by one-way ANOVA and *t* test analyses.

### Data availability.

The 117 MAGs retrieved in this study are available in the NCBI database under accession numbers JAABPS000000000 to JAABUE000000000 under BioProject number PRJNA594844.
