# Supplementary figures and images for: Compositional and Metabolic Responses of Autotrophic Microbial Community to Salinity in Lacustrine Environments
Source: mSystems. 2022 Jul 12;7(4):e00335-22. doi: 10.1128/msystems.00335-22 (PMC9426519; doi:10.1128/msystems.00335-22)

a

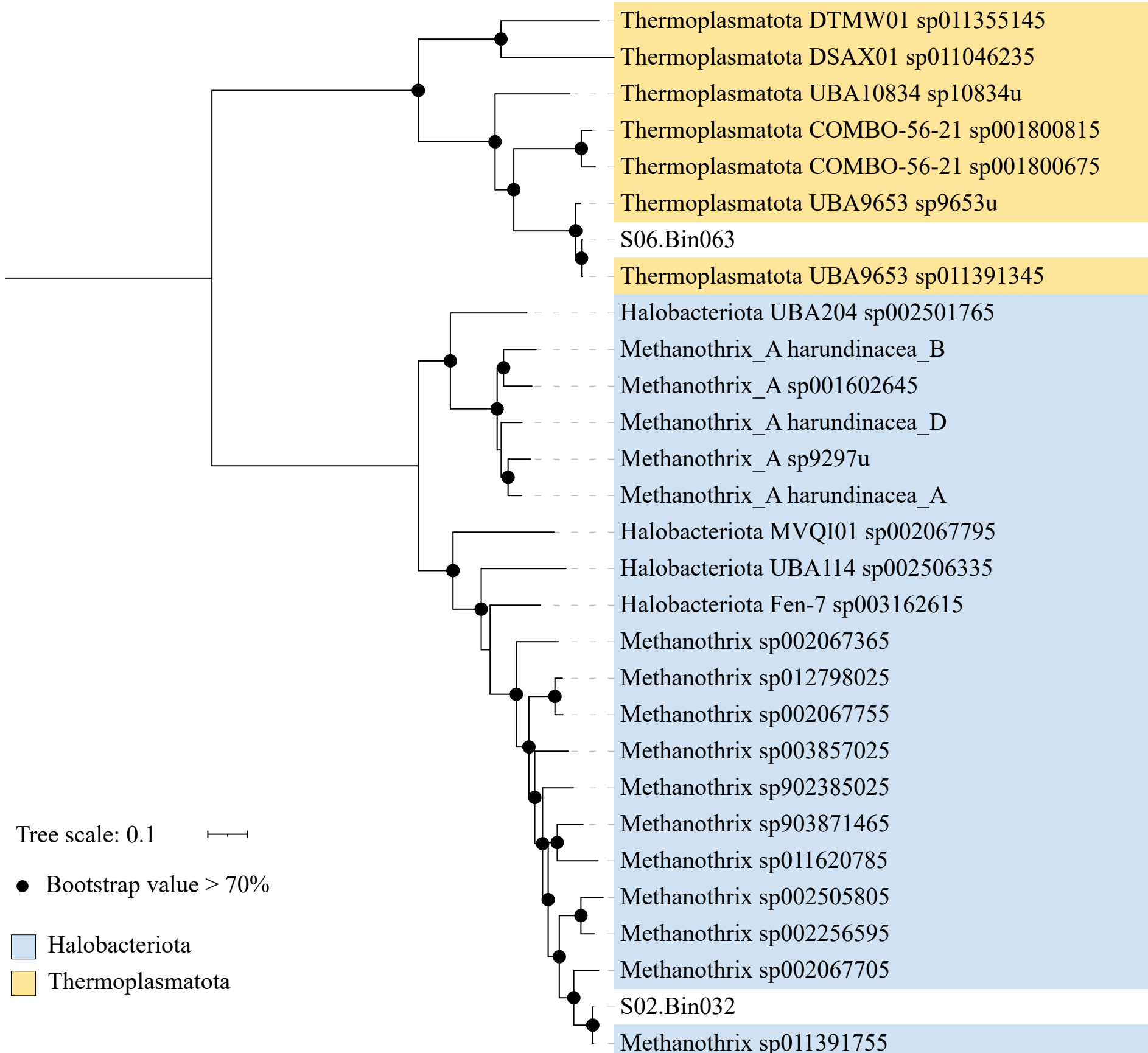

b

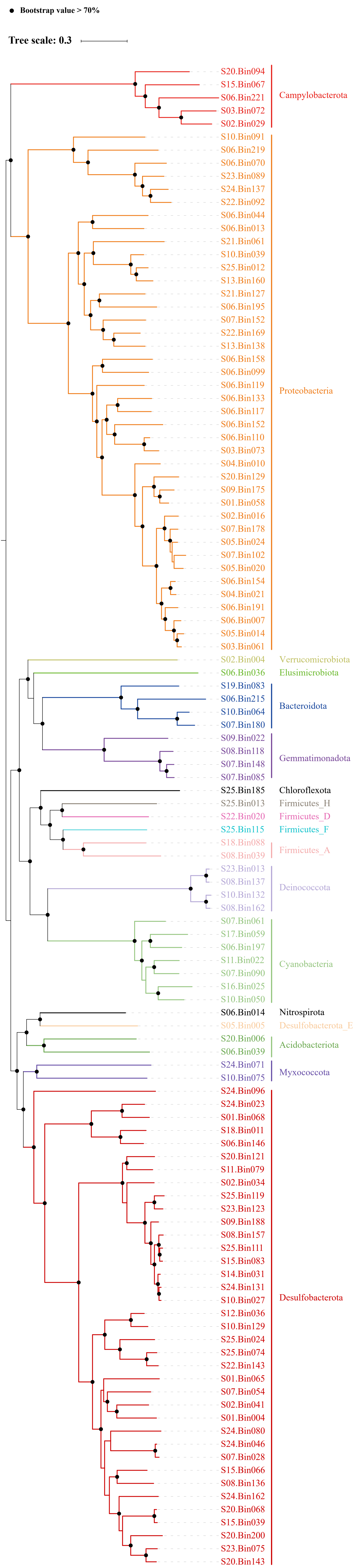

Supplement: FIG S1 [file msystems.00335-22-s0001.pdf]

a

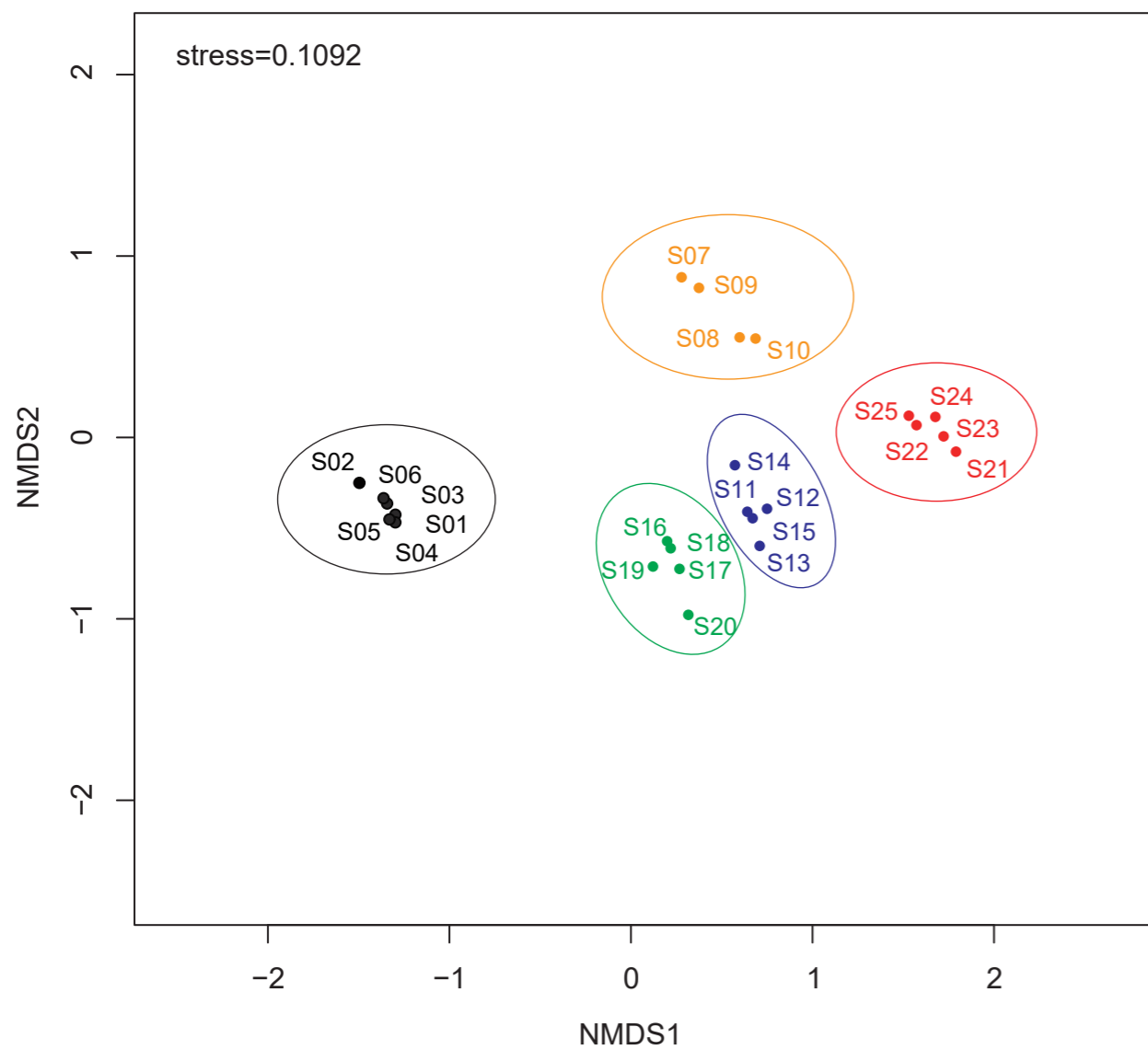

b

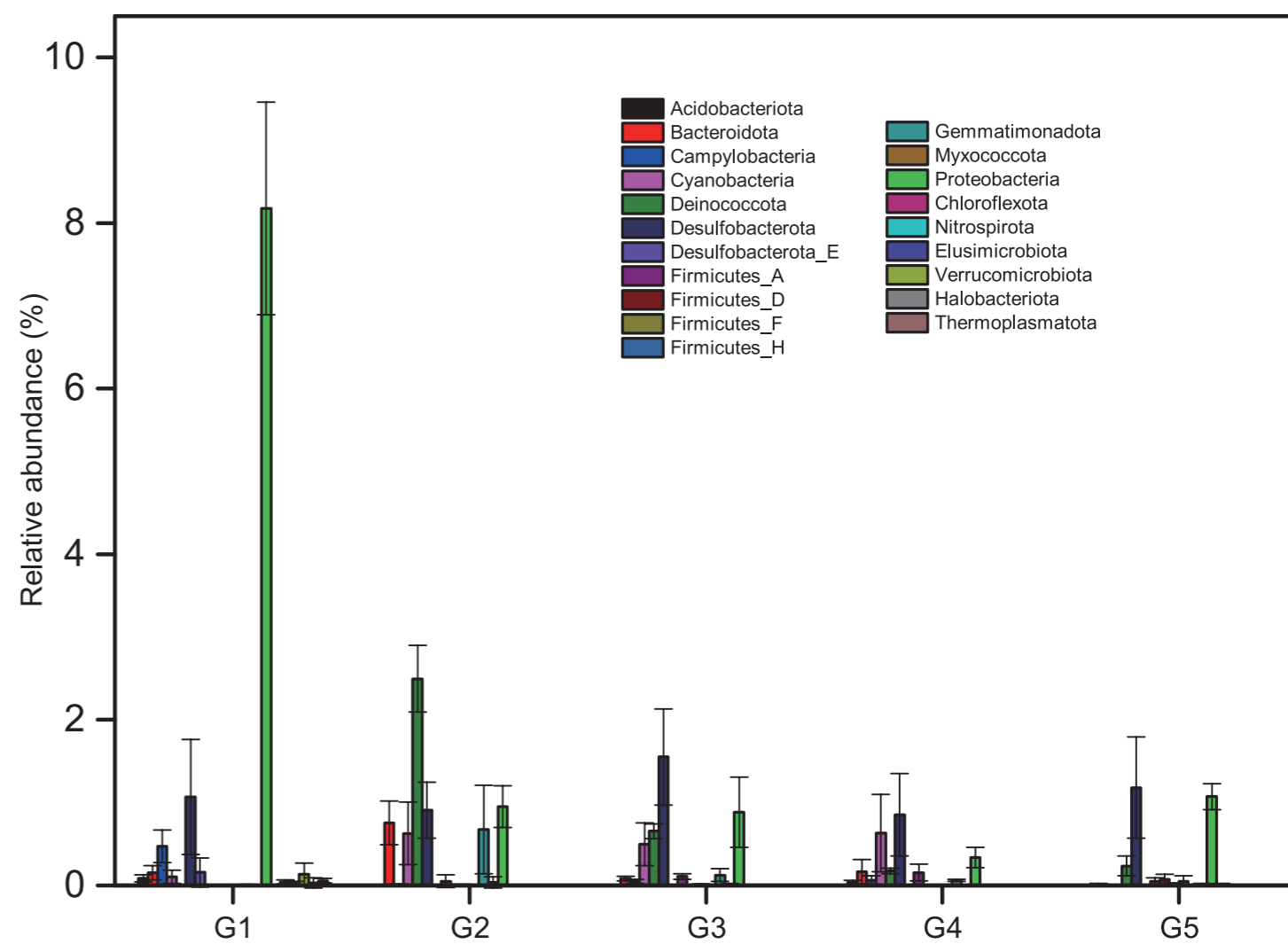

c

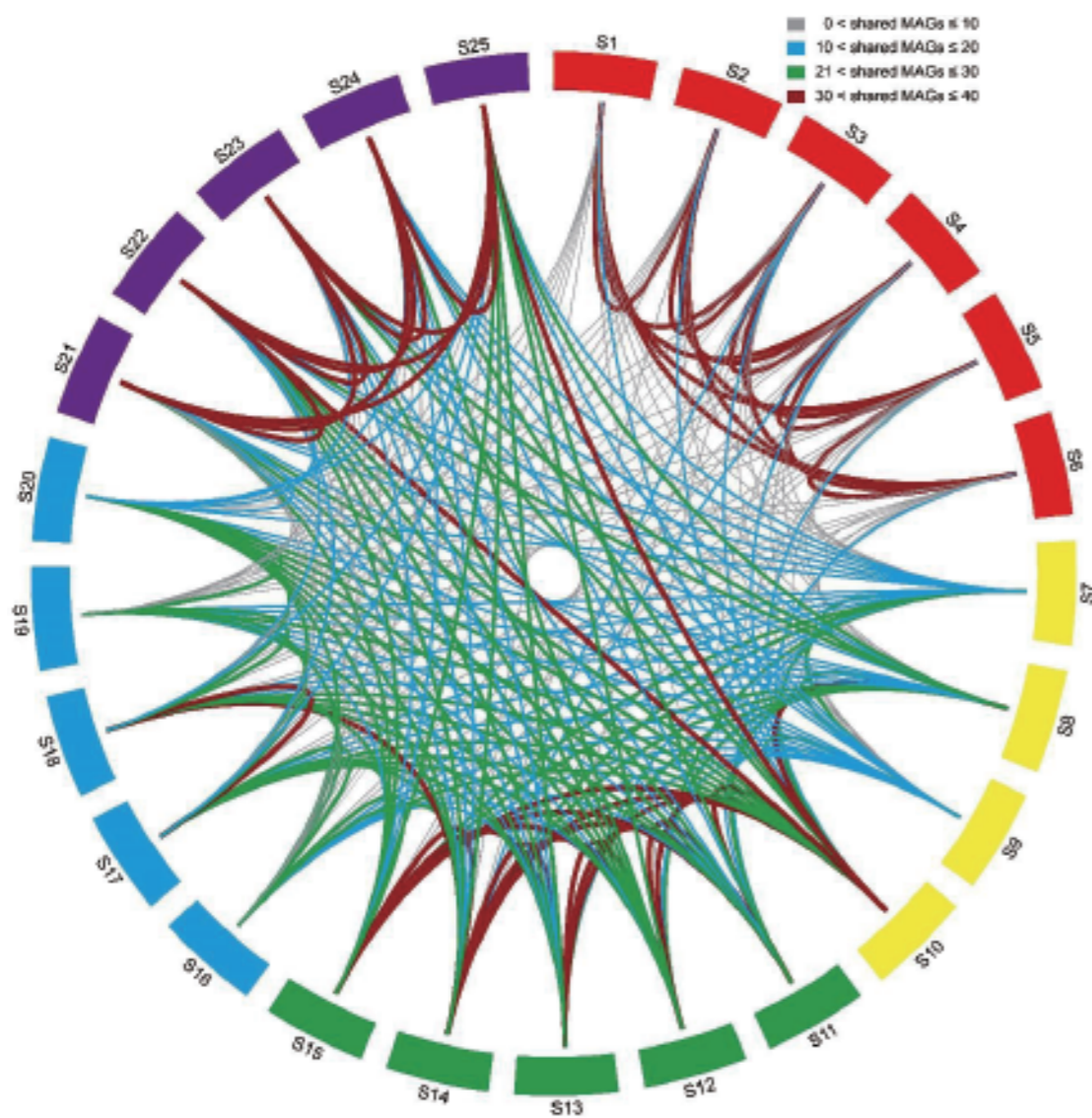

Supplement: FIG S2 [file msystems.00335-22-s0002.pdf]

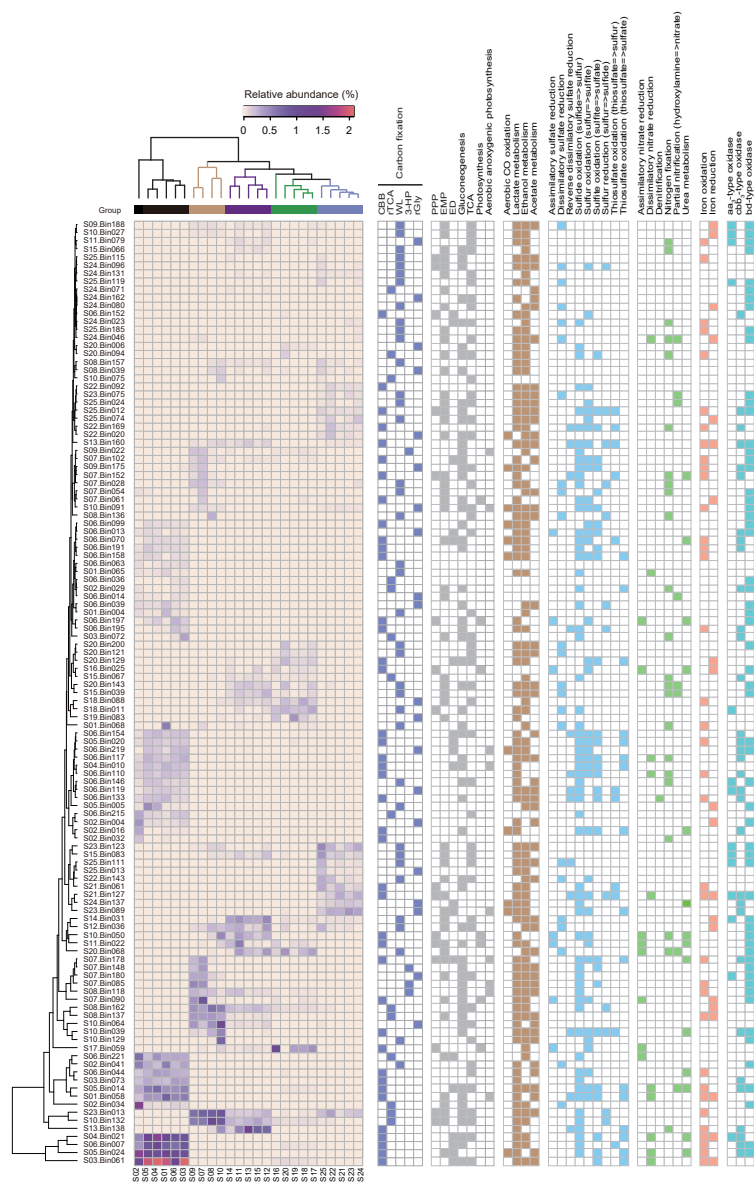

Supplement: FIG S3 [file msystems.00335-22-s0003.pdf]

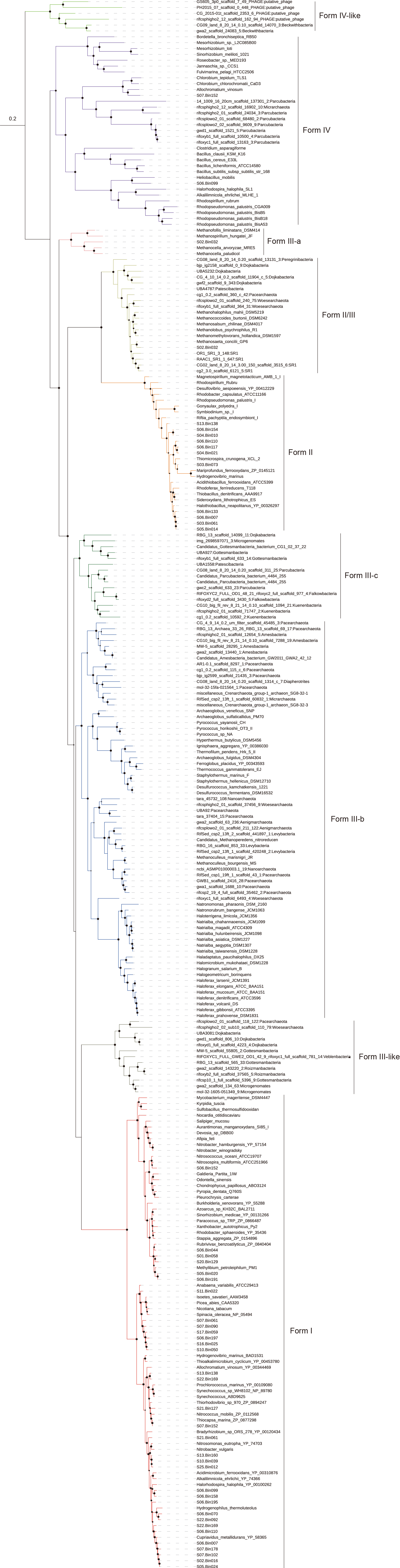

Supplement: FIG S4 [file msystems.00335-22-s0004.jpg]

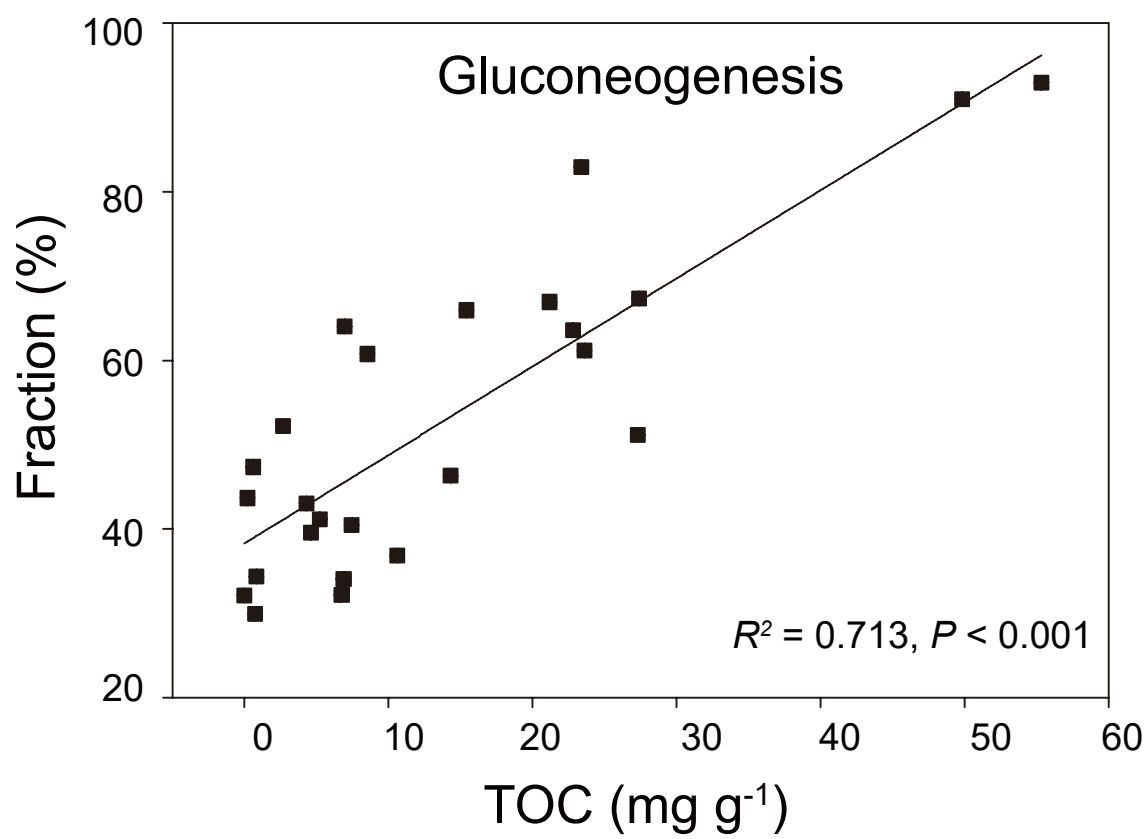

Supplement: FIG S5 [file msystems.00335-22-s0005.pdf]

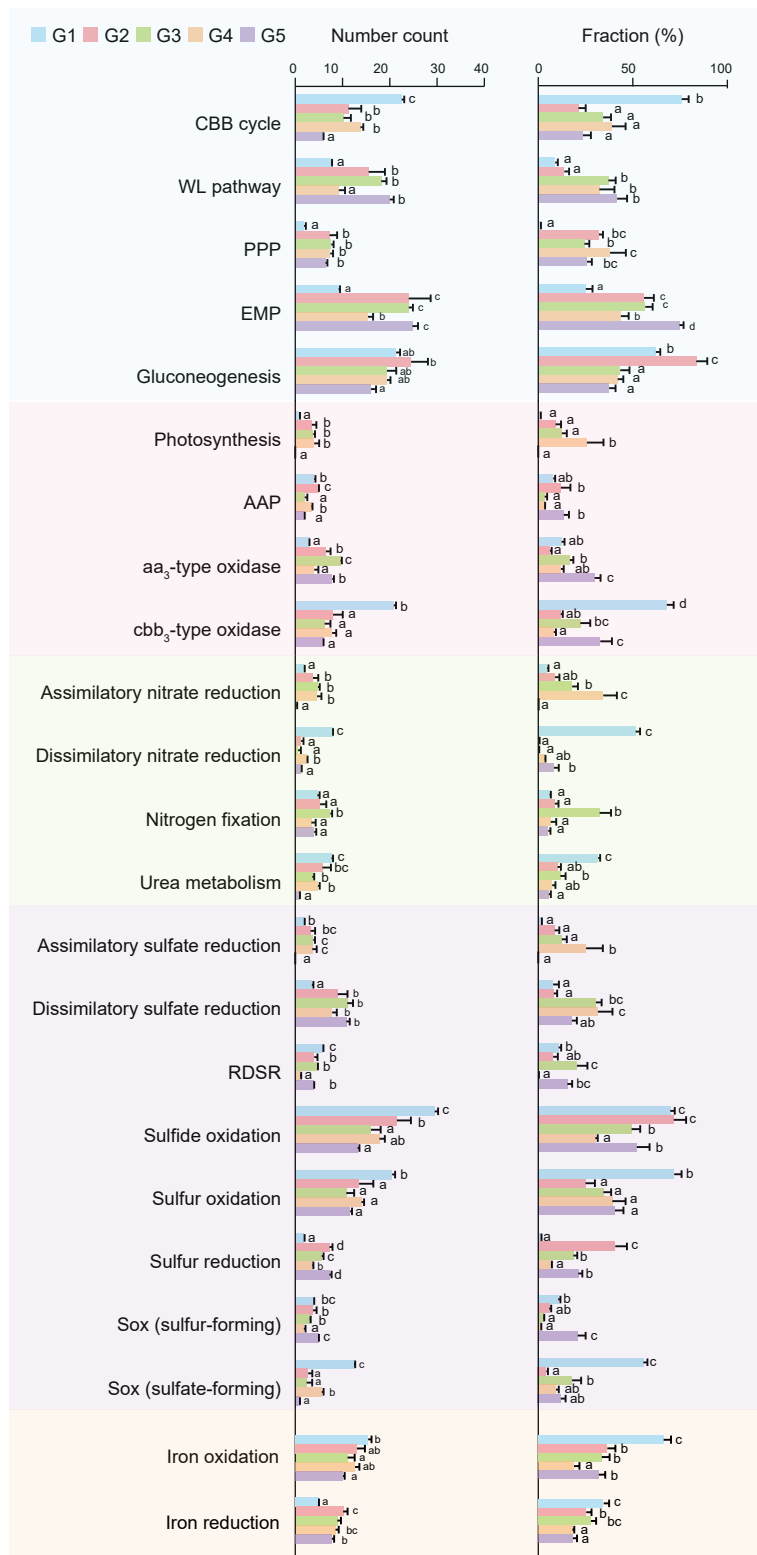

Supplement: FIG S7 [file msystems.00335-22-s0007.pdf]
