# Supplementary material for: Compositional and Metabolic Responses of Autotrophic Microbial Community to Salinity in Lacustrine Environments
Source: mSystems. 2022 Jul 12;7(4):e00335-22. doi: 10.1128/msystems.00335-22 (PMC9426519; doi:10.1128/msystems.00335-22)
Supplement: TEXT S1 [file msystems.00335-22-s0009.docx]

Supplementary Information

**Compositional and metabolic responses of autotrophic microbial community to salinity in lacustrine environments**

**Methods**

**Relative abundance calculation**

Community composition was determined using the ribosomal protein S3 (rpS3) as previously reported (1). A total of 29,644 rpS3 were identified in 25 sediment samples using AMPHORA2 (2), and then were clustered at 99% with USEARCH. Read mapping of all 25 individual samples was performed using BBMap with the parameters “minid = 0.97, local = t”. BlastP of the rpS3 clusters against the rpS3 identified on the 117 non-redundant genomes was carried out. The relative abundance of each bin was calculated by dividing the coverage of its rpS3 by the total coverage of all rpS3 in the community. For the genomes without rpS3 identified, another method was used to calculate their relative abundances across samples. In brief, scaffold coverages of the 117 genomes with carbon fixation potential for each sample were calculated by mapping qualified reads against these scaffolds with BBMap and the script jgi_summarize_bam_contig_depths in MetaBAT 2 (3), and then genome coverage was estimated by averaging all scaffold coverages of specific genome in one sample. Subsequently, relationships between genome coverages and relative abundances of these bins possessing rpS3 were represented by equations (e.g., linear, quadratic, power and logarithm), which were used to estimate relative abundances of the bins without rpS3 identified.

**References**

1. Anantharaman K, Brown CT, Hug LA, Sharon I, Castelle CJ, Probst AJ, Thomas BC, Singh A, Wilkins MJ, Karaoz U, Brodie EL. 2016. Thousands of microbial genomes shed light on interconnected biogeochemical processes in an aquifer system. Nature Commun 7:1-1.
2. Wu M, Scott AJ. 2012. Phylogenomic analysis of bacterial and archaeal sequences with AMPHORA2. Bioinformatics 28:1033-1034.
3. Kang D, Li F, Kirton ES, Thomas A, Egan RS, An H, Wang Z. 2019. MetaBAT 2: an adaptive binning algorithm for robust and efficient genome reconstruction from metagenome assemblies. Peer J 7:e27522v1.
