# Supplementary material for: Compositional and Metabolic Responses of Autotrophic Microbial Community to Salinity in Lacustrine Environments
Source: mSystems. 2022 Jul 12;7(4):e00335-22. doi: 10.1128/msystems.00335-22 (PMC9426519; doi:10.1128/msystems.00335-22)
Supplement: FIG S6 [file msystems.00335-22-s0006.pdf]

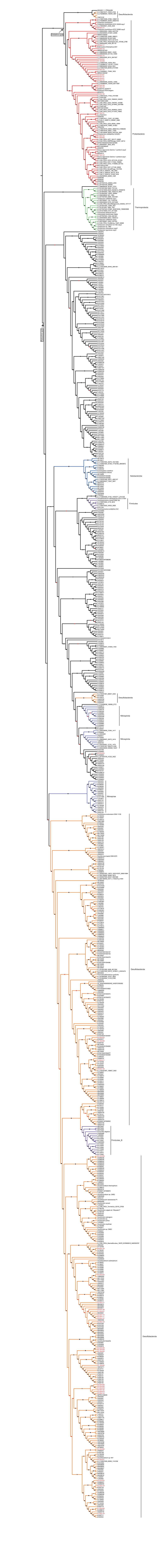
Chlorobi
Desulfobacterota
Proteobacteria
Thermoproteota
Halobacteriota
Firmicutes
Desulfobacterota
Nitrospirota
Nitrospirota
Nitrospirae
Desulfobacterota
Firmicutes_B
Desulfobacterota
